# Inactivation efficacy and mechanisms of atmospheric cold plasma on *Alicyclobacillus acidoterrestris*: Insight into the influence of growth temperature on survival

**DOI:** 10.3389/fnut.2022.1012901

**Published:** 2022-09-15

**Authors:** Lang-Hong Wang, Lin Chen, Siqi Zhao, Yanyan Huang, Xin-An Zeng, Rana Muhammad Aadil

**Affiliations:** ^1^School of Food Science and Engineering, Guangdong Provincial Key Laboratory of Intelligent Food Manufacturing, Foshan University, Foshan, China; ^2^College of Food Science and Technology and College of Life Sciences, Northwest University, Xi'an, China; ^3^School of Food Science and Engineering, South China University of Technology, Guangzhou, China; ^4^National Institute of Food Science and Technology, University of Agriculture, Faisalabad, Pakistan

**Keywords:** *Alicyclobacillus acidoterrestris*, cold plasma, scanning electron microscopy, FT-IR, GC-MS

## Abstract

The bactericidal effect of dielectric barrier discharge-atmospheric cold plasma (DBD-ACP, 20, and 30 kV) against *Alicyclobacillus acidoterrestris* on the saline solution and apple juice was investigated. Results show that DBD-ACP is effective for the inactivation of *A. acidoterrestris* by causing significant changes in cell membrane permeability and bacterial morphology. The effect of culture temperatures on the resistance of *A. acidoterrestris* to DBD-ACP was also studied. *A. acidoterrestris* cells grown at 25°C had the lowest resistance but it was gradually increased as the culture temperature was increased (25–45°C) (*p* < 0.05). Moreover, results from Fourier transform infrared spectroscopy (FT-IR) and Gas Chromatography-Mass Spectrometer (GC-MS) analysis showed that the increase in the culture temperature can gradually cause the decreased level of cyclohexaneundecanoic acid in the cell membrane of *A. acidoterrestris* (*p* < 0.05). In contrast, cyclopentaneundecanoic acid, palmitic acid, and stearic acid showed an increasing trend in which the fluidity of the bacterial cell membrane decreased. This study shows a specific correlation between the resistance of *A. acidoterrestris* and the fatty acid composition of the cell membrane to DBD-ACP.

## Introduction

*Alicyclobacillus acidoterrestris* is a kind of spore-producing acidophilic heat-resistant bacteria. Many studies have shown that this bacterium is one of the main reasons for causing the spoilage of various fruit juices such as apple juice, orange juice, and grape juice (1–3). The fruit juice contaminated with *A. acidoterrestris* did not show obvious rancidity or swelling at the initial stage. However, if its metabolites, (2, 6-dibromophenol, and 2, 6-dibromophenol) produced by *A. acidoterrestris* cells can cause quality issues by increasing turbidity as well as formation of white precipitates in the juice. These losses can cause problems for fruit juices by causing huge financial losses (4). Keeping in mind the potential risk to the quality of fruit and its beverages, *A. acidoterrestris* has been proposed as a reference microorganism for quality control in the juice industry. Heat pasteurization is the most common method in fruit juice processing. However, due to the acidophilic and heat-resistant characteristics of *A. acidoterrestris* cells, it is difficult to inactivate by heat pasteurization. Moreover, thermal processing could easily cause flavor components and heat-sensitive nutrients such as vitamins that significantly damage fruit juice (5). Therefore, *A. acidoterrestris* is an actual problem that urgently needs to be solved in fruit juice processing. It is essential to seek a safe, reliable, and effective means to kill *A. acidoterrestris* with less influence on fruit juice components.

Nonthermal plasma technology is an aggregate formed by atoms, electrons, charged particles, free radicals, and ultraviolet photons generated by ionized or partially ionized gases (6–8). Cold plasm can inactivate various microbial vegetative cells in food at low temperatures (generally lower than 40°C) and short time, but it has little effect on the quality of the food itself. So it has been considered as a potential technology for sterilization of various types of food (9). Studies have shown that many factors including gas composition, voltage and treatment time, and food characteristics affect the efficiency of cold plasma sterilization. Additionally, the inherent characteristics, including cell structure and intrinsic protective mechanisms of microorganisms and environmental factors, are also essential factors (10, 11). Temperature and pH are two of the common factors that can induce changes in the resistance of bacteria to decontamination processing, including plasma, which in turn affects sterilization efficiency (7, 12–14). However, from the results reported so far, the studies about temperature-mediated bacterial tolerance to plasma are obscure and insufficient (14, 15). Because of the potential hazard of *A. acidoterrestris* in the juice industry, it is of great significance to fully understand the bactericidal effect and influencing factors of non-thermal plasma on *A. acidoterrestris* to promote the application of this technology in juice sterilization. Therefore, this work is mainly aimed to investigate the bactericidal effect and mechanism for the inactivation of *A. acidoterrestris* cells by dielectric barrier discharge-atmospheric cold plasma (DBD-ACP). The changes in cell membrane permeability and morphology of *A. acidoterrestris* cells before and after DBD-ACP treatment were evaluated by an inverted fluorescence microscope, intracellular leakage contents, and scanning electron microscope. The effects of growth temperatures on the resistance of *A. acidoterrestris* cells to DBD-ACP were also studied based on cell membrane fluidity and membrane fatty acid composition from Fourier transform infrared spectroscopy (FT-IR) and gas-mass spectrometry. This work is expected to provide insight into the inactivation properties, mechanism, and resistance under various growth temperatures of *A. acidoterrestris* cells induced by DBD-ACP.

## Materials and methods

### Bacterial cultures

The bacterial strain of *A. acidoterrestris* ATCC 49025 was purchased as a lyophilized culture from the Microbial Culture Collection Center of Guangdong Institute of Microbiology (Guangzhou, China). *A. acidoterrestris* cells were revived by transferring the lyophilized culture to an AAM liquid medium (pH 4.0, per liter of deionized water containing 0.2 g ammonia sulfate, 0.25 g calcium chloride, 0.5 g magnesium sulfate, 2.0 g yeast extract, 5.0 g glucose, and 3.0 g monopotassium phosphate) and incubated overnight at 45°C in a shaker (200 rpm). A loopful of the culture of *A. acidoterrestris* cells suspension was inoculated onto cooled AAM agar medium and incubated at 45°C for 36 h. Then a single colony was transferred to a 500 mL-conical flask containing 200 mL of sterile AAM liquid medium and incubated to a late log phase of growth at 45°C. Pre-cultured *A. acidoterrestris* cells were transferred to a fresh AAM liquid medium (200 mL, OD 600 nm ≈ 0.10) for incubation at 25, 35, 45 and 55 °C.

### DBD-plasma treatment

The cultured *A. acidoterrestris* was harvested by centrifugation at 4,000 × g for 10 min at 4°C. The collected pellet was washed with sterile water thrice and resuspended into sterilized saline water or apple juice (JinLiuYuan, not from concentration). It was prepared to be treated with DBD-ACP (CTP-2000K plasma equipment, equipped with DBD-50 reactor, Nanjing Suman Co., Ltd. Nanjing, China) at 20 kV and 30 kV for 0, 0.5, 1.0, 1.5 and 2.0 min, where the distance between the upper plate and liquid surface was 4 mm, and the frequency was 1.0 kHz. The treated bacterial solution was serially diluted, and the spread plate was counted, and cultured at 45°C for 36–48.0 h to detect the total number of colonies (CFU/mL). The lethality was analyzed according to the change in the number of colonies before (*N*_0_) and after (*N*) DBD-ACP treatment. Each treatment was repeated three times and the experimental results were averaged.

### Cell membrane permeability of *A. Acidoterrestris*

*A. acidoterrestris* cells were subjected to DBD-ACP treatment under 30 kV for 0, 0.5, 1.0 and 2.0 min. After DBD-ACP treatment, 10 μL of *A. acidoterrestris* cells suspension was added to 990 μL of sterilized saline water containing 3.0 μL of propidium iodide (PI, 1 mg mL^−1^, Beijing Soleibao Technology Co., Ltd., Beijing, China) and incubated darkly at room temperature for 20 min. The red fluorescence of *A. acidoterrestris* cells from PI staining was observed by an inverted fluorescence microscope (Nikon Eclipse Ti2-A, Nikon Instruments Co. LTD., Tokyo, Japan). Additionally, membrane permeabilization was also evaluated by measuring the leakage of nucleic acids including DNA and RNA, and proteins from *A. acidoterrestris* cells using a NanoDrop spectrophotometer (ND-2000, Thermo Fisher Scientific, Massachusetts, USA) to record the absorbance at 260 nm and 280 nm according to the method reported by Cai et al. (16).

### Scanning electron microscopy

Bacterial sample preparation for morphological observation was carried out regarding the previous method (17). *A. acidoterrestris* cells were collected by centrifuging at 4,000 × *g* for 5.0 min and then treated with 2.5% (v/v) glutaraldehyde in PBS buffer were stored overnight at 4°C. After centrifugation, the bacterial samples were dehydrated by gradient using 30–100% ethanol solution. The dehydrated cells were treated with tert-butanol twice. The samples were dropped on silver paper for vacuum freeze-drying, and the morphological changes of *A. acidoterrestris* cells could be observed after spraying gold.

### Cell membrane fatty acid extraction and analysis

Fatty acid composition of *A. acidoterrestris* cells was detected using the method previously reported by Pan et al. (18). After membrane fatty acid extraction and methylation, the fatty acid profiles were detected by gas chromatography-mass spectrometry (GC-MS) (8890B−7000D, Agilent Technologies, Palo Alto, California, USA) by matching the mass spectra with the mass spectral library 2016 of the National Institute of Standards and Technology (NIST, Gaithersburg, Maryland, USA) and the retention times in the bacterial acid methyl ester (BAME) mix solution (analytical standard, Sigma-Supelco, Bellefonte, PA, USA).

### Fourier transform infrared spectroscopy analysis

The lyophilized *A. acidoterrestris* cells were analyzed by infrared spectrum on Bruker Vetex70 FT-IR spectrometer (Bruker, Germany). The spectrum collection range was 600–4,000 cm^−1^ and the resolution was 4 cm^−1^. The spectral data is smoothed, normalized and converted to a second derivative to improve peak resolution using the Savtitzky-Golay algorithm (19).

### Statistical analyses

Statistical analysis was performed using OriginPro 8.0 (Origin Lab, Northampton, MA, USA) in triplicate with three independent experiments and results expressed as means ± SD. Analysis of variance (ANOVA) followed by Tukey's test was carried out using SPSS 22.0 software (IBM, NY, USA), and values were considered significantly different if *p* < 0.05.

## Results and discussion

### Inactivation of *A. Acidoterrestris* cells by DBD-ACP

**Figure 1:**
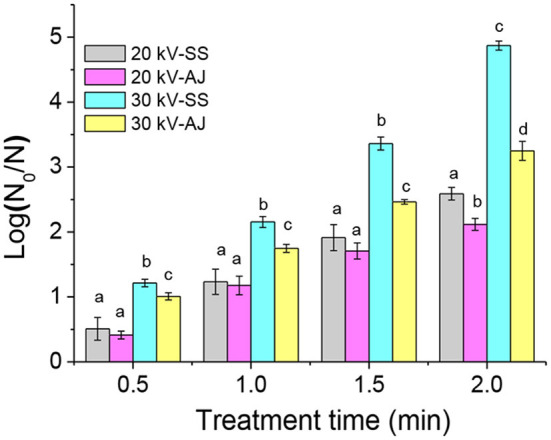
Inactivation of *A. acidoterrestris* by DBD-plasma at 20 kV and 30 kV on apple juice (AJ) and 0.85% sterile saline solution (SS). Different letters (a–d) on the top of the bars denote significant differences (*p* < 0.05).

The inactivation efficacy of DBD-ACP under various treatment voltage and time against *A. acidoterrestris* cells in 0.85% sterile saline solution and apple juice is shown in Figure 1. For *A. acidoterrestris* cells in 0.85% sterile saline solution, 0.5 and 1.2-log reductions occurred for the exposure of DBD-ACP at 20 kV for 0.5 and 1 min and increased to approximately 1.9- and 2.5-log reductions for 1.5 and 2 min. Comparatively, DBD-ACP exhibited a much stronger bactericidal effect on *A. acidoterrestris* cells at 30 kV, which induces the bacterial populations was inactivated by approximately 1.2, 2.1, 3.4 and 4.9-log reductions with the same treatment time.

A similar trend of DBD-ACP inactivation was detected for *A. acidoterrestris* cells in apple juice. However, DBD-ACP has a relatively lower inactivation against *A. acidoterrestris* cells in apple juice than in sterile saline solution. A similar work conducted by Mahnot et al. (20), found that *S. enterica serovar Typhimurium* in distilled water and the inactivation (>5 log-reduction) was observed much higher than in other simulating tender coconut water. Moreover, they also suggested that the presence of phosphate and Mg^2+^ ions reduced the inactivation of *S. enterica serovar Typhimurium*. Therefore, it could be inferred that the presence of nutrients and ions in apple juice may help to repair and recover *A. acidoterrestris* cells after DBD-ACP treatment.

### Membrane permeability of *A. Acidoterrestris* cells

**Figure 2:**
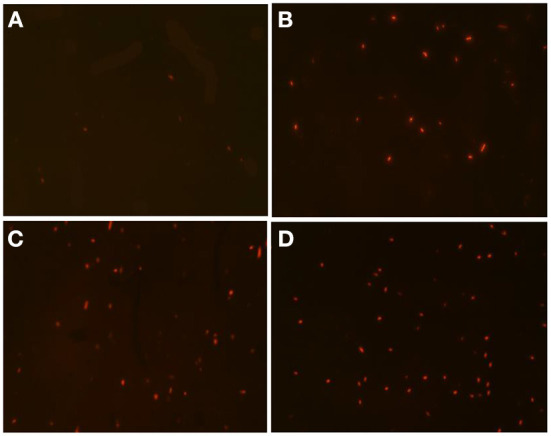
The PI staining *A. acidoterrestris* cells treated by DBD-plasma at 30 kV for 0 **(A)**, 0.5 min **(B)**, 1.0 min **(C)**, and 2.0 min **(D)**, respectively.

The cell membrane permeability of *A. acidoterrestris* cells after DBD-ACP treatment was observed by staining with PI, which incorporates genomic DNA by emitting red fluorescence if the bacterial cell membrane is permeable (21). As shown in Figure 2A, *A. acidoterrestris* cells without DBD-ACP treatment did not show much red fluorescence. Whereas the DBD-ACP treated *A. acidoterrestris* was stained with PI to emit higher degrees of red fluorescence with increasing time (Figures 2B–D). These results indicated that the plasma-treated *A. acidoterrestris* cell membrane was damaged, resulting in an increased permeability.

**Figure 3:**
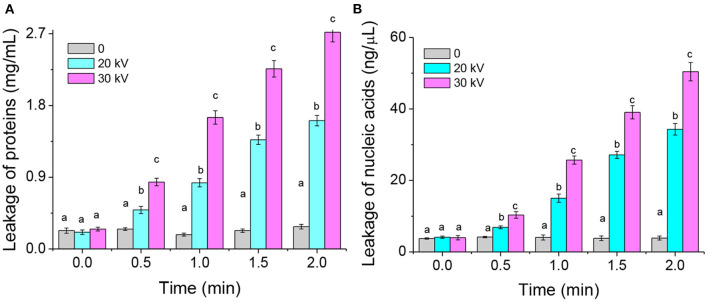
Leakage of proteins **(A)** and nucleic acids **(B)** from *A. acidoterrestris* cells induced by DBD-ACP. Different letters (a-d) on the top of the bars denote significant differences (*p* < 0.05).

The cell membrane permeability induced by DBD-ACP may increase the leakage of cytoplasmic contents, including nucleic acids and proteins. Thus, the leakage of cytoplasmic contents of *A. acidoterrestris* cells was further determined in this study. The results of protein leakage of the DBD-ACP treated and untreated bacteria are shown in Figure 3A. The protein content in the untreated bacteria group was about 0.25 mg/L. However, this leakage of protein increases to 1.61 and 2.72 mg/L after being treated by DBD-ACP at 20 and 30 kV for 2.0 min. The nucleic acid concentration of *A. acidoterrestris* cells with or without DBD-ACP treatment presented a similar behavior (Figure 3B). As compared to untreated cells, the nucleic acid concentration was increased from 4.12 and 3.98 ng/μL to 34.31 and 50.42 ng/μL for *A. acidoterrestris* cells treated by DBD-ACP at 20 and 30 kV for 2.0 min, respectively. These results were consistent with the results obtained by PI staining with an inverted fluorescence microscope. Qian et al. (2022) found that the leakage of nucleic acids and proteins of *L. monocytogenes* and *S. enteritidis* induced reached the maximum content by cold plasma at 90 and 150 s, respectively (22). Olatunde et al. (23) reported a loss in cell membrane integrity of bacteria, including *L. monocytogenes, S. aureus, P. aeruginosa, E. coli*, and *V. parahaemolyticus*, which was measured by the increased conductivity and DNA content in supernatant induced by DBD-ACP treatment. In conclusion, the changes in membrane permeability that causes the formation of reversible permeabilization in bacterial cells were responsible for the lethal damage of *A. acidoterrestris* after DBD-ACP treatment.

### Morphological changes of *A. Acidoterrestris* cells induced by DBD-ACP

**Figure 4:**
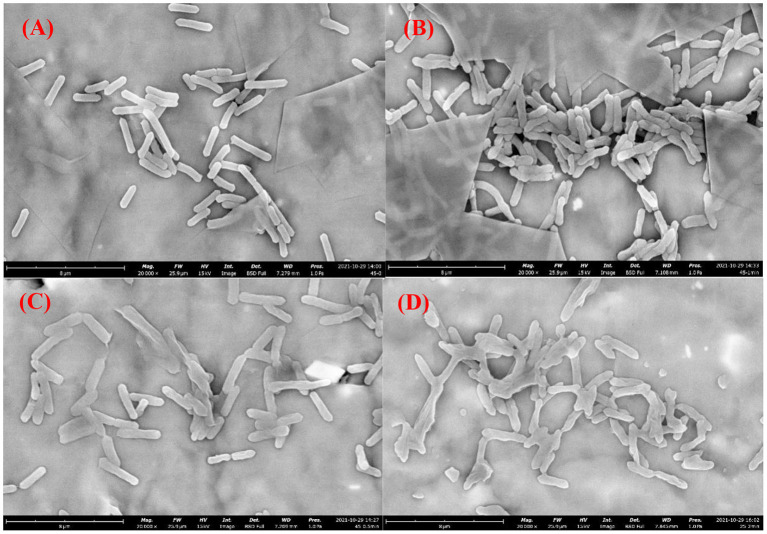
Morphological images of *Alicyclobacillus acidoterrestris* cells growth at 45°C exposure to DBD-plasma at 30 kV for 0 **(A)**, 0.5 **(B)**, 1.0 **(C)** and 2.0 min **(D)**, respectively.

Figures 4A–D show the cell morphological changes of *A. acidoterrestris* cells before and after DBD-ACP treatment at the voltage of 30 kV for 0.5, 1.0 and 2.0 min. Results showed that the untreated *A. acidoterrestris* cells showed a short rod-like shape with a smooth surface, while the DBD-ACP treated cells showed wrinkles, shrinkage, and deformation on the surface. These results suggested that *A. acidoterrestris* cells exhibit significant changes in their morphology with the increasing treatment. Such cell morphological deformations have been observed in *E. coli, L. monocytogenes*, and *S. enteritidis* after cold plasma treatment (24–26). Thus, it could be inferred that the lethal effect of *A. acidoterrestris* cell could be attributed to its destructive effects on the bacterial membrane with the leakage of cytoplasmic components by reactive oxygen species (ROS), particularly O_3_ and atomic oxygen generated during the processing of DBD-ACP treatment.

### Effect of growth temperature on the resistance of *A. Acidoterrestris* to DBD-ACP

**Figure 5:**
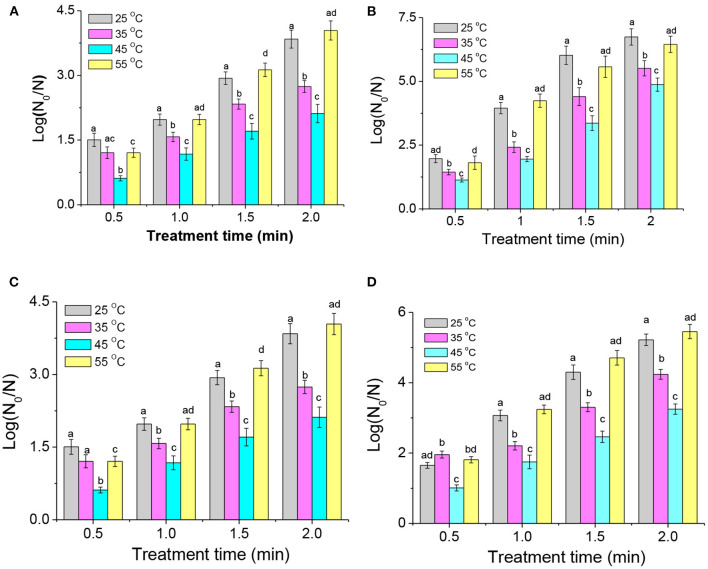
The effect of growth temperatures on the resistance of *A. acidoterrestris* cells on the sterile saline solution to DBA-ACP under 20 kV **(A)** and 30 kV **(B)**, and apple juice with 20 kV **(C)** and 30 kV **(D)** for different treatment times. Different letters (a–d) on the top of the bars denote significant differences (*p* < 0.05).

The temperature, pH, and other growth conditions play a vital role in bacterial resistance against inactivation technologies such as thermal, acidic, high-pressure, and pulsed electric field treatments. After being grown at 25, 35, 45, and 55°C to the late-logarithmic phase, *A. acidoterrestris* cells were collected and re-suspended on different systems (sterilized saline solution and apple juice) for DBD-ACP treatment. As shown in Figure 5, the inactivation of *A. acidoterrestris* cells increases with the increasing treatment voltage and time. On the sterilized saline solution, the inactivation levels of *A. acidoterrestris* cells cultivated at 25°C that increased from 1.8 to 3.8 log-reduction and from 1.9 to 6.7 log-reduction for DBD-ACP at 20 kV and 30 kV with treatment time from 0.5 to 2.0 min. Bacterial cells at different growth temperatures exhibited different resistance to DBD-ACP treatment. Among them, *A. acidoterrestris* cells grown at 45°C were found to be the most resistant, followed by at 35°C. Interestingly, the resistance of *A. acidoterrestris* cells at 25 and 55°C were similar (*p* > 0.05), and more sensitive to DBD-ACP than at 45 and 35°C no matter on the sterilized saline solution (Figures 5A,B) and apple juice (Figures 5C,D).

For the sterilized saline solution, DBD-ACP treatment at the voltage of 20 kV with 1.0 min resulted in a 2.3 and 2.4 log-reduction (*p*>0.05) of *A. acidoterrestris* cultivated at 25 and 55°C, respectively. In contrast, the degree of inactivation of *A. acidoterrestris* was significantly lower by 1.6 log-reduction and (1.0 logs) (*p* < 0.05) for being cultivated at 35°C and 45°C, respectively. As the treatment time increased to 2.0 min, the inactivation of *A. acidoterrestris* cultured at different temperatures increased significantly. However, the inactivation behavior of *A. acidoterrestris* by DBD-ACP treatment was still similar. Specifically, the inactivation of *A. acidoterrestris* grown at 55°C was the highest that reaching to 5.3 log-reduction after DBD-ACP treatment, followed by *A. acidoterrestris* cultured at 25°C (4.8 log-reductions), and the inactivation rate was significantly decreased to 3.4 and 2.6 log-reduction for cells grown at 35 and 45°C, respectively (*p* < 0.05). Additionally, when the treatment voltage was 30 kV with the same treatment time of 2.0 min (Figure 5B), then the inactivation of *A. acidoterrestris* cultured at 25 and 55°C reached 6.7 and 6.4 log-reduction, respectively. The amount of inactivation at 35 and 45°C was significantly reduced (*p* < 0.05) to 5.5 and 4.9 log-reduction, respectively. The effect of growth temperatures on *A. acidoterrestris* cells exposed to DBD-ACP treatment with apple juice has similar results. For example, *A. acidoterrestris* cells grown at 25 and 55°C, DBD-ACP induced 5.2 and 5.4 log-reductions at 30 kV for 2 min, while *A. acidoterrestris* cells cultivated at 35 and 45°C resulted in 4.2 and 3.2 log reductions of viability, respectively. These results suggest that the resistance of *A. acidoterrestris* to DBD-ACP gradually increased with the increase of culture temperature in the range of 25 ~ 45°C expect 55°C. The above results concerning the effect of growth temperature on plasma inactivation are inconsistent with the resistance data reported by Pan et al. (27) revealing that the inactivation of *L. monocytogenes* cultivated at 10°C was found to be the most resistant to plasma exposure. Additionally, Fernandez et al. (15) found that the effect of growth temperatures (20, 25, 37, and 45°C) on the inactivation efficiency of *Salmonella enterica serovar Typhimurium* by cold plasma was insignificant (15). This inconsistency may be due to the different bacteria owning various adaptive mechanisms to temperature, resulting in different consequences regarding the effect of growth temperature on the resistance to DBD-ACP. However, the present study was similar to the literature showing that bacteria such as *E. coli, L. monocytogenes*, and *S. aureus* inoculated at low cultured temperatures showed high sentences to thermal or some nonthermal processing techniques including pulsed electrical field, high-pressure processing and atmosphere uniform glow discharge plasma (13, 14, 28). For example, Kayes et al. (14) found that *E. coli, S. aureus*, and *B. subtilis* cultured at 35°C were more tolerant to air plasma than bacteria cultured at 10°C (14).

### Analysis of the cell membrane fluidity

The distinct difference in resistance to DBD-ACP may be responsible for the alterations of cell membrane fluidity and fatty acid composition of *A. acidoterrestris* at the different growth temperatures. The cellular membrane is one of the main targets being attacked by ROS and free radicals induced by cold plasma. As membrane fluidity is distinctive for the exchange of nutrients, ions, and regulatory molecules of cells, which directly affects the permeation of ROS into bacterial cells (7, 29). Therefore, the membrane fluidity was worth investigating.

**Figure 6:**
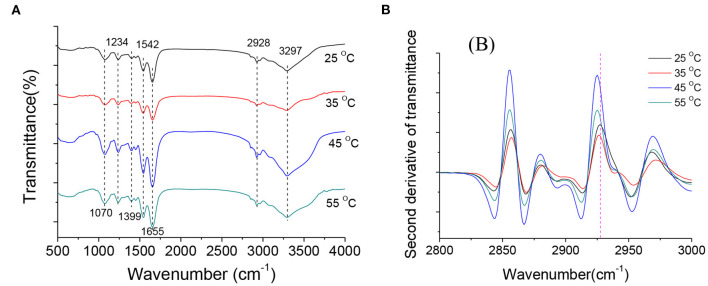
Raw FT-IR spectra of *A. acidoterrestris* at late log phase under different growth temperatures **(A)** and calculated second derivatives of FT-IR spectra in the spectral range of 2,800–3,000 cm^−1^ **(B)**.

FT-IR is a sensitive, non-destructive technique commonly used to detect changes in bacterial cell composition (30, 31). In Figure 6A, the FT-IR spectra of *A. acidoterrestris* cultured at different temperatures showed some typical characteristic peaks: 1,070 cm^−1^ and 1,234 cm^−1^ are typical P = O asymmetric and symmetric stretching vibration peaks, representing the main chain of nucleic acid and phosphodiester, respectively; while 1,399 cm^−1^ is the asymmetric and symmetric deformation of CH_3−_ and CH_2−_ of the protein; 1,542 cm^−1^ is the N-H characteristic vibration peak of protein amide II band; 1,655 cm^−1^ is the absorption peak near 1 is mainly the vibration peak of protein amide I, which is assigned to C=O stretching vibration; 2,928 cm^−1^ is the asymmetric stretching vibration peak of ω-cyclic fatty acid acyl chain υsCH_2−_. The above results in agreement with the previous study (32), indicating that the basic structure of the bacteria cultured at different temperatures is consistent. The differences in the infrared spectra exhibited by *A. acidoterrestris* at different temperatures are not noticeable.

To further study the changes in fatty acid and fluidity of the cell membrane of *A. acidoterrestris* at different temperatures. In this study, the 2,800 ~ 3,000 cm^−1^ infrared spectral data were processed by the second derivative to enhance spectral resolution. It can be seen from Figure 6B that the spectra of this band at different culture temperatures show specific differences, indicating that different culture temperatures have certain effects on the composition of fatty acids in the cell membrane of *A. acidoterrestris*. According to the asymmetric stretching vibration of ω-cyclic fatty acid acyl chain υsCH_2−_ near 2,928 cm^−1^, the changes in cell membrane fluidity at different temperatures were analyzed, and it was found that when the culture temperature increased from 25 to 55°C, the peak frequency from 2,927 cm^−1^ drops to 2,923 cm^−1^. Previous studies suggested that the peak frequency of fatty acid acyl chain υsCH_2_- is related to cell membrane fluidity. It is generally believed that the larger the value, the greater the fluidity of the cell membrane, and *vice versa* (19). Thus, it can be inferred that the membrane fluidity of *A. acidoterrestris* cells decreased gradually with the increase in culture temperature.

### Membranes fatty acid analysis of *A. Acidoterrestris* Cells

Previously, studies have shown that bacteria at different temperatures and pH alter membrane fatty acid composition in order to maintain a proper cell membrane fluidity and to ensure the normal functioning of membrane physiology (33, 34). For example, the saturated fatty acid content in *Shewanella putrefaciens* cell decreased with the decrease of incubation temperature (30, 10 and 4°C), while the content of palmitoleic acid (C16:1), lauric acid (C12:0), and myristic acid (C14:0) increased (33). Similarly, *E. coli* cells were apt to increase the proportions of saturated fatty acids at the expense of unsaturated fatty acids, as reported by Liu et al. (28), who found that the proportion of unsaturated fatty acids decreased from 51.63 to 29.58% as the growth temperature increased from 15 to 45°C. In a recent study, *A. acidoterrestris* was found to increase the level of cyclic fatty acids with the expense of saturated fatty acids to improve the acid-tolerance capability (3).

The membrane fatty acid composition of the cell of *A. acidoterrestris* cultured at different temperatures is shown in Table 1. There are five main types of fatty acids detected, namely myristic acid (C14:0), palmitic acid (C16:0), stearic acid (C18:0), cyclohexaneundecanoic acid (ω-cyclohexyl C17:0) and cyclopentanetridecanoic acid (ω-cyclopentane C18:0), which has good consistency with the results obtained by Zhao et al. (3). In this study, *A. acidoterrestris* as an acidophilic heat-resistant bacterium and ω-cyclohexyl C17:0 and ω-cyclopentane C18:0 accounted for more than 80%, which is good in consistence with previous studies that ω-cyclic fatty acids appear in the cell membrane of acidophilic, heat-resistant bacteria, in which the proportion could reach 60 to 90% (35, 36). The fatty acid components including C16:0, C18:0, ω-cyclohexyl C17:0, and ω-cyclopentane C18:0 showed a significant change under various culture temperatures. Among them, the relative content of ω-cyclohexyl C17:0 was the highest when the culture temperature was 25°C, accounting for 81.9%. With the increase in culture temperature (35-55°C), the proportion of ω-cyclohexyl C17:0 was gradually decreased, accounting for 72.37, 59.23, and 49.86%, respectively (*p* < 0.05). In contrast, the level of ω-cyclopentane C18:0 showed an upward trend, increasing from 10.83 to 32.21% with an increased temperature of 25 to 55°C, respectively. In a study, as compared with straight-chain fatty acids, cyclic fatty acids can reduce the *trans-gauche* isomerization of lipid chains, thereby increasing the order degree of lipid chains and the area of lipid layers, and reducing the fluidity of cell membranes (37, 38). However, a study has shown that the loose packing of ω-alicyclic fatty acids in the cell membrane is in the disorder of lipid tails increasing membrane fluidity, and the larger the ring size (3- to 7-membered cycloalkyl) in ω-alicyclic fatty acids, the disorder degree is greater (36). On the other hand, the relative proportion of C16:0 and C18:0 also increased, i.e., from 4.36, 2.43% at 25°C and to 11.35 and 5.85%, at 55°C. Generally, straight-chain fatty acids, including C16:0 and C18:0 are neatly arranged in the cell membrane, and the stacking is good and tight. Therefore, the higher the proportion of straight-chain fatty acids in the cell membrane, the lower the fluidity of the cell membrane (17, 39, 40). Thus, it could be inferred that the decreased cell membrane fluidity with the increased culture temperature of *A. acidoterrestris* cells are the results of an increase of C16:0, C18:0, and ω-cyclopentane C18:0.

**Table 1:** The proportions of primary membrane fatty acid composition of *A. acidoterrestris* under different growth temperatures.

**Fatty acid composition (%)**	**Growth temperature**			
	**25°C**	**35°C**	**45°C**	**55°C**
C14:0	1.21 ± 0.05a	1.27 ± 0.07a	1.23 ± 0.12a	0.66 ± 0.09a
C16:0	4.36 ± 0.52a	5.79 ± 0.84b	8.19 ± 0.98c	11.35 ± 1.49d
C18:0	2.42 ± 0.49a	3.61 ± 0.33b	4.53 ± 0.88bc	5.85 ± 1.48c
ω-cyclohexyl C17:0	81.09 ± 1.23a	72.37 ± 1.63b	59.23 ± 1.53c	49.86 ± 1.83d
ω-cyclopentane C18:0	10.83 ± 1.53a	16.94 ± 1.81b	26.81 ± 1.47c	32.21 ± 1.86d
SFAs	7.99 ± 1.06a	10.67 ± 1.24ab	13.95 ± 1.98c	17.86 ± 3.06d
CFAs	91.92 ± 2.76a	89.31 ± 3.44ab	86.04 ± 3.0b	82.07 ± 3.69bc

Values followed by a different letter (a–d) in the same line are significantly different (p < 0.05).

### Implication for the resistance of *A. Acidoterrestris* cells with membrane fatty acid profile

The stacked loose ω-alicyclic fatty acids can protect acidophilus from high temperature and low pH conditions by forming a dynamic barrier that restricts lipid diffusion and H^+^ transmembrane diffusion (36). However, different from the mechanism of high temperature and low acid action, various reactive oxygen species and reactive nitrogen components such as ozone, hydroxyl radicals, and singlet oxygen radicals generated by plasma treatment, which will damage the function of fatty acids, induce lipids, especially the peroxidation of unsaturated fatty acids even destroys the cell membrane and leads to cell lysis and bacterial death (25, 41). In addition, the free radicals generated by plasma treatment can also cross the cell membrane and enter the cytoplasm to attack the biological macromolecules such as nucleic acids (25). In this study, *A. acidoterrestris* as an acidophilic heat-resistant bacteria, and ω-cyclohexyl C17:0 and ω-cyclopentane C18:0 accounted for more than 80%. With the increase in culture temperature (25–55°C), the total content of straight-chain fatty acids in the cell membrane of *A. acidoterrestris* increased gradually from 8.0 to 17.86% (*p* <0.05). In contrast, the content of cyclic fatty acids decreased from 92.92 to 82.07%. It can be inferred that loosely stacked ω-alicyclic fatty acids (especially ω-cyclohexyl C17:0) may be more conducive to the accumulation of oxygen free radicals and other reactive components in the cell membrane, that not only cause damage to the cell membrane but also increases oxygen free radicals and other components entry and cause cell death. In contrast, the well-arranged, packed, tight straight-chained fatty acids may prevent the entry of these harmful substances to a certain extent. Therefore, the decrease of cyclic fatty acid content and the increase of straight-chain fatty acids caused by the increase in culture temperature may be the reason for the increase in plasma tolerance of *A. acidoterrestris* in the culture temperature range of 25 to 45°C. It is worth mentioning that the content of straight-chain fatty acids in the cell membrane of *A. acidoterrestris* was the highest when cultured at 55°C, but these cells are highly sensitive to DBD-ACP treatment. These results may be related to the aerobic metabolism of *A. acidoterrestris* was improved at higher temperatures (55°C). Reportedly, the generation of intracellular reactive oxygen species by aerobic metabolism was increased with environment temperature (42). Therefore, it could speculate that a growth temperature of 55°C might cause remarkable effects on the plasma-mediated accumulated abundance of intracellular ROS inducing bacteria to be more sensitive to DBD-ACP treatment (7, 43).

## Conclusions

The inactivation of DBD-ACP on *A. acidoterrestris* and effects of growth temperatures on their resistance was investigated. Results show that DBD-ACP is effective in the inactivation of *A. acidoterrestris* by causing a significant increase in cell membrane permeability with leakage of cytoplasmic contents and changes in bacterial morphology. *A. acidoterrestris* cells at different growth temperatures (25, 35 45 and 55°C) exhibited different resistance to DBD-ACP treatment. *A. acidoterrestris* cells grown at 45°C were found to be the most resistant, followed by at 35°C, 25°C and 55°C. The growth temperatures of *A. acidoterrestris* induced noticeable modifications of membrane fatty acid profile and fluidity during cultivation. For example, the most abundant fatty acids, i.e. ω-cyclohexyl C17:0 and ω-cyclopentane C18:0, changed from 81.09% to 49.86% and 10.83% to 32.21% at the growth temperatures of 25 to 55°C. Growth temperature-mediated alterations in fatty acid profile and membrane fluidity of *A. acidoterrestris* were associated with its viability to DBD plasma exposure and a significant increase in resistance with increased growth temperatures from 25 to 45°C. It is worth mentioning that *A. acidoterrestris* cells grown at 55°C are highly sensitive to DBD-ACP treatment. These results may be related to the growth temperature of 55°C causing remarkable effects on the plasma-mediated accumulated abundance of intracellular ROS inducing bacteria to be more sensitive to DBD-ACP treatment. In conclusion, this study demonstrated DBD-ACP is a promising technology in inactivating *A. acidoterrestris*, but it is worth noting growth temperature plays a role in the resistance of *A. acidoterrestris* to DBD-ACP, suggesting a potential way to improve the sterilization efficiency in practical application by adjusting the preconditioning temperature. However, more investigations are still needed to further understand how *A. acidoterrestris* regulate membrane fatty acid and fluidity based on the genetics and metabolomics in response to various temperature.

## Data availability statement

The original contributions presented in the study are included in the article/supplementary material, further inquiries can be directed to the corresponding authors.

## Author contributions

L-HW: conceptualization, methodology, software, investigation, and writing—original draft. LC: methodology, validation, formal analysis, visualization, and data curation. SZ and RA: writing—review & editing. YH: resources, methodology, and supervision. X-AZ: resources, writing—review & editing, supervision, and data curation. All authors contributed to the article and approved the submitted version.

## Funding

Support for this research by the Project of Science and Technology Department of Shaanxi Province (2021JQ-448), the Key Laboratory Project of Guangdong Province (2022B1212010015), and the National Natural Science Foundation of China (3210160758) are all gratefully acknowledged.

## Conflict of interest

The authors declare that the research was conducted in the absence of any commercial or financial relationships that could be construed as a potential conflict of interest.

## Publisher's note

All claims expressed in this article are solely those of the authors and do not necessarily represent those of their affiliated organizations, or those of the publisher, the editors and the reviewers. Any product that may be evaluated in this article, or claim that may be made by its manufacturer, is not guaranteed or endorsed by the publisher.
